# Crystal structure and Hirshfeld surface analysis of the 1:3 adduct of tetra­aqua­trinitrato­neodymium(III) with 3-amino-1,2,4-triazine

**DOI:** 10.1107/S2056989018011714

**Published:** 2018-08-24

**Authors:** Ramalingam Sangeetha, Kasthuri Balasubramani, Savaridasson Jose Kavitha, Madhukumar Hemamalini

**Affiliations:** a Department of Chemistry, Government Arts College(Autonomous), Karur 639 005, Tamil Nadu, India; b Department of Chemistry, Mother Teresa Womens University, Kodaikanal 624 102, Tamil Nadu, India

**Keywords:** crystal structure, adduct, triazine, neodymium(III), three-dimensional supra­molecular hydrogen bond, Hirshfeld surface analysis

## Abstract

In the title compound, [Nd(NO_3_)_3_(H_2_O)_4_]·3C_3_H_4_N_4_, neodymium is ten-coordinate with a distorted bicapped square-anti­prismatic geometry formed from six O atoms from three nitrate ions and four O atoms from four coordinated water mol­ecules. The structure also contains neutral 3-amino-1,2,4-triazine mol­ecules which are not coordinated to the central metal atom. The coordinated water mol­ecules and nitrate ions of adjacent complexes are linked by O—H⋯O hydrogen bonds to form cyclic *R*_2_^2^(8) ring motifs, which in turn are further connected *via* hydrogen bonds to generate a sheet-like structure. The triazine mol­ecules are involved in a number of hydrogen-bonding inter­actions: N—H⋯N and O—H⋯N inter­actions to form *R*_3_^3^(9) motifs and N—H⋯N inter­actions to link the organic mol­ecules into chains. Weak C—H⋯O hydrogen bonds also occur between triazine mol­ecules and coordinated nitrate atoms. All these inter­molecular contacts contribute to the stabilization of the three-dimensional supra­molecular framework. Hirshfeld surface analysis shows that N⋯H/H⋯N and H⋯H inter­actions account for 42.9 and 20.6% of the surface, respectively.

## Abstract 2

The asymmetric unit of the adduct contains a neodymium(III) cation, three coordinated nitrate anions, four coordinated water mol­ecules and three uncoordinated neutraltriazine mol­ecules. The crystal structure consists of a three-dimensional supra­molecular framework held together by a network of O—H⋯O and O—H⋯N hydrogen bonds between the coordinated water mol­ecules, nitrate ions and triazine mol­ecules. The uncoordinated neutral triazine moieties form N—H⋯N hydrogen bonds. Hirshfeld surface and fingerprint plots identify the major contributors to the inter­molecular inter­actions.

## Chemical context   

Lanthanide complexes with organic ligands have many applications related to the design and synthesis of potential anti­cancer and anti­bacterial agents (Eliseeva & Bunzli, 2010 ▸; Liu *et al.*, 2008 ▸; Kostova & Stefanova, 2009 ▸; Siddiqi *et al.*, 2009 ▸; Taha *et al.*, 2011 ▸; Hermann *et al.*, 2008 ▸; Gassner *et al.*, 2008 ▸; Xu *et al.*, 2010 ▸). Some lanthanide complexes also have potential roles in the treatment of malignant cells (Kostova *et al.*, 2004 ▸). In addition, coordination polymers of lanthanide ions have been investigated for use as sensors, catalysts and MRI contrast agents and in applications in the areas of magnetism, gas absorption, self-assembly and medicine (Li *et al.*, 2015 ▸; Bunzli *et al.*, 2015 ▸; Wang *et al.*, 2016 ▸; Zhang & Lin, 2014 ▸).

Triazine heterocyclic π-conjugated structures are attractive organic mol­ecules owing to the chemical flexibility of their systems and have many applications in medicinal chemistry, materials science and organic synthesis (Boesveld & Lappert, 1997 ▸; Boesveld *et al.*, 1999 ▸; Reid *et al.*, 2011 ▸). Triazine deriv­atives have been used as building blocks for subtle chemical architectures comprising organic–inorganic hybrid frameworks (Ma­thias *et al.*, 1994 ▸; Zerkowski & Whitesides, 1994 ▸; MacDonald & Whitesides, 1994 ▸; Guru Row, 1999 ▸; Krische & Lehn, 2000 ▸; Sherrington & Taskinen, 2001 ▸). We report herein the crystal structure of a new lanthanide complex with 3-amino-1,2,4-triazine.
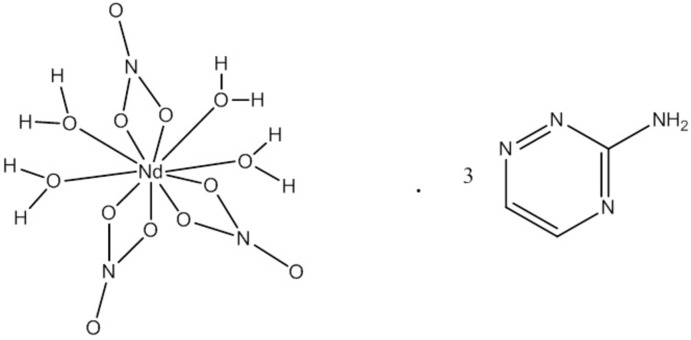


## Structural commentary   

**Figure 1:**
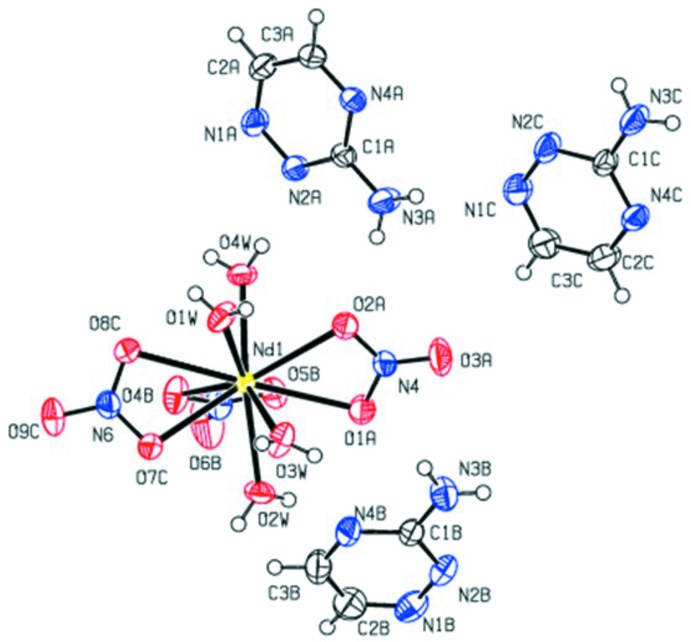
The asymmetric unit of the title compound with the atom-numbering scheme. Displacement ellipsoids for non-H atoms are drawn at the 50% probability level.

The asymmetric unit of the title compound (Fig. 1 ▸) contains a neodymium(III) cation, three coordinated nitrate anions, four coordinated water mol­ecules and three uncoordinated neutral 3-amino-1,2,4-triazine mol­ecules. The Nd^III^ ion is ten coordinate and has a distorted bicapped square-anti­prismatic geometry, being surrounded by six oxygen atoms from three nitrate ions and four oxygen atoms from coordinated water mol­ecules. The lengths of the Ni—O bonds (Table 1 ▸) are in good agreement with those reported in the literature (Trzesowska-Kruszynska *et al.*, 2010 ▸).

## Supra­molecular features   

**Figure 2:**
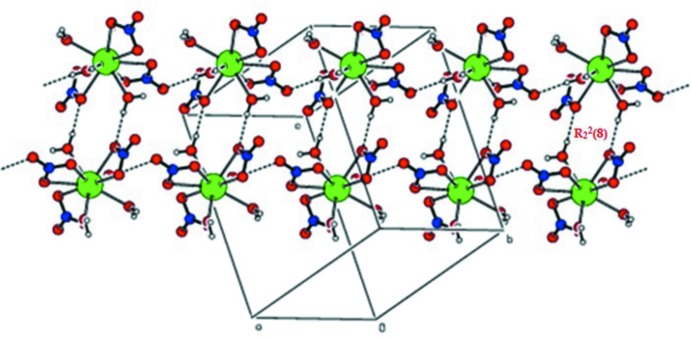
A view of the O—H⋯O hydrogen-bonding inter­actions (shown as dotted lines) between coordinated water mol­ecules and nitrate ions, which generate a sheet-like structure.

**Figure 3:**
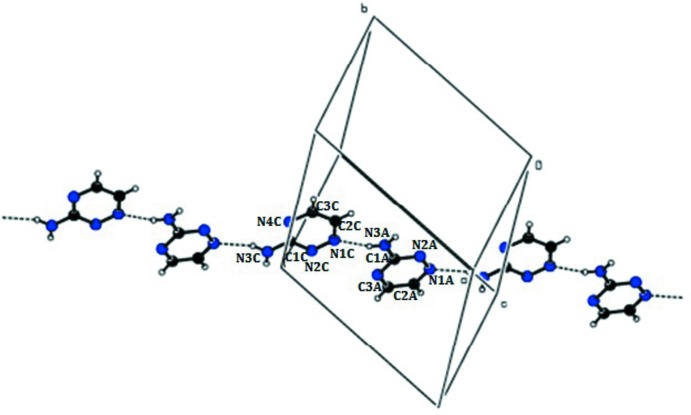
A view of N—H⋯N hydrogen-bonded pairs (shown as dotted lines) between triazine moieties (*A* and *C*) extending into zigzag chains.

**Figure 4:**
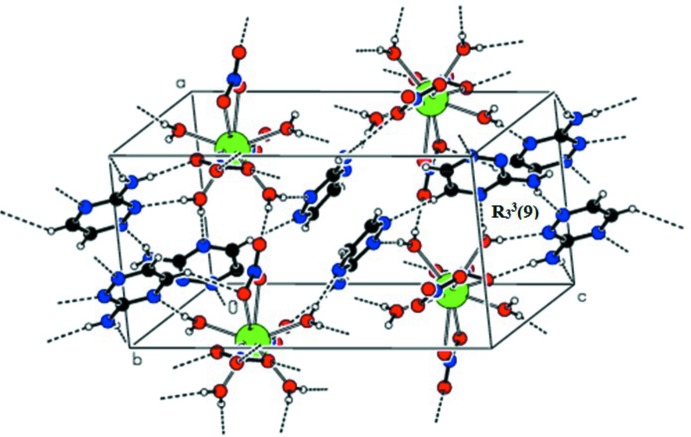
An overall view of the three-dimensional supra­molecular framework of the title compound.

In the crystal, the coordinated water mol­ecules act as hydrogen-atom donors (Table 2 ▸) to the oxygen atoms of nitrate ions in adjacent mol­ecules and are linked by a set of O—H⋯O [O2*W*—H2*A*⋯O6*B* ^iii^ and O3*W*—H3*A*⋯O7*C* ^v^] hydrogen bonds, forming cyclic 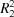
(8) ring motifs. These ring motifs are further connected *via* O—H⋯O hydrogen bonds to generate a sheet-like structure (Fig. 2 ▸). The uncoordinated neutral triazine moieties (*A* & *C*) are connected *via* N—H⋯N [N3*C*—H2*NC*⋯N1*A* ^vii^ and N3*A*—H2*NA*⋯N1*C* ^iii^] hydrogen bonds, forming zigzag chains (Fig. 3 ▸). The triazine mol­ecules are also involved in N—H⋯N and O—H⋯N hydrogen-bonding inter­actions, forming 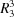
(9) motifs (Fig. 4 ▸). The carbon-bound hydrogen atoms of the triazine moieties (*B* & *C*) are linked through weak C—H⋯O [C3*B*—H3*BA*⋯O6*B* ^ix^ and C3*C*—H3*CA*⋯O4*B* ^vii^] hydrogen bonds formed with the coordinated nitrate atoms (*B*). All these inter­molecular inter­actions appear to play a significant role in stabilizing the crystal structure and result in the formation of a three-dimensional supra­molecular framework (Fig. 4 ▸).

## Hirshfeld surface analysis   

**Figure 5:**
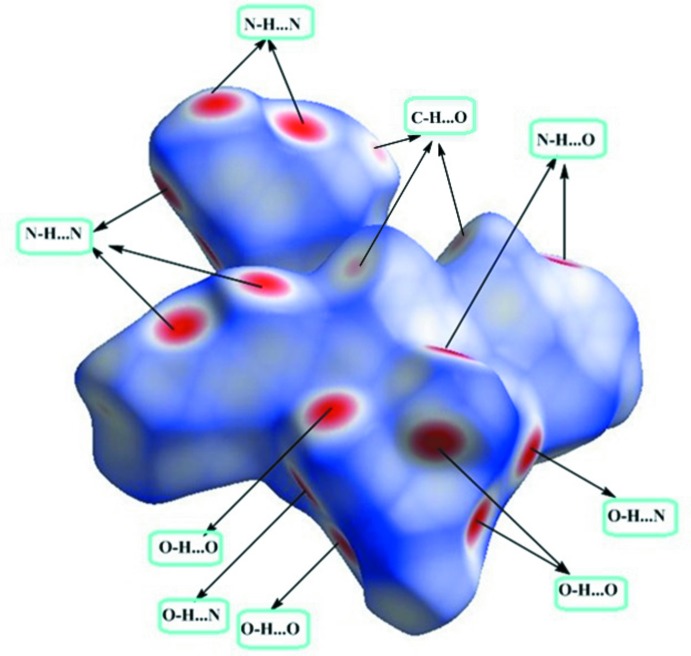
Three-dimensional Hirshfeld surfaces of the title compound plotted over *d*_norm_.

**Figure 6:**
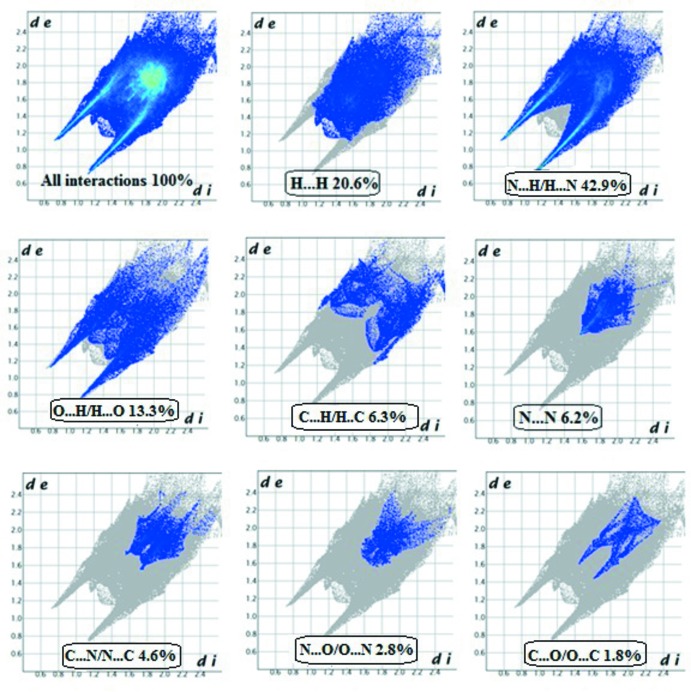
Two-dimensional fingerprint plots of the title compound showing the contributions of the different inter­actions. *d*_e_ and *d*_i_ represent the distances from a point on the Hirshfeld surface to the nearest atoms outside (external) and inside (inter­nal) the surface, respectively.

Hirshfeld surface analysis (Spackman & Jayatilaka, 2009 ▸) and two-dimensional fingerprint plots, which are useful tools for describing the surface characteristics of the crystal structure, were generated using *CrystalExplorer3.0* (Wolff *et al.*, 2012 ▸). The normalized contact distance (*d*_norm_) is based on the distances from the nearest atom inside (*d*_i_) and outside (*d*_e_) the surface. The three-dimensional *d*_norm_ surface of the title compound is shown in Fig. 5 ▸. The red points represent short contacts and negative *d*_norm_ values on the surface correspond to the N—H⋯N, N—H⋯O and O—H⋯O inter­actions. Analysis of the two-dimensional fingerprint plots reveal that the H⋯H (20.6%) and N⋯H/H⋯N (42.9%) inter­actions are the highest contributors to the Hirshfeld surface. Smaller contributions come from O⋯H/H⋯O (13.3%) C⋯H/H⋯C (6.3%), N⋯N (6.2%), C⋯N/N⋯C (4.6%), N⋯O/O⋯N (2.8%) and C⋯O/O⋯C (1.8%) inter­actions (Fig. 6 ▸).

## Database survey   

A search of the Cambridge Structural Database (Version 5.39, update February 2018; Groom *et al.*, 2016 ▸) for 3-amino-1,2,4-triazine yielded four structures crystallizing as metal com­plexes: KUCNAY [with bis­(3-amino-1,2,4-triazine-*N* ^2^)-bis(hexa­fluoro­acetyl­acetonato-*O*,*O*′)copper(II)] and KUCNEC [with bis­(μ 2-3-amino-1,2,4-triazine-*N* ^1^,*N* ^4^)hexa­kis­(hexafluoro­acetyl­acetonato-*O*,*O*′)tricopper(II)] (Li *et al.*, 2009 ▸); WOZXOA {with *catena*-[bis­(μ_2_-dicyanamido)­bis­(1,2,4-triazin-3-amine)­cobalt]; Palion-Gazda *et al.*, 2015 ▸} and WOZXOA01 {with *catena*-[bis­(μ_2_-dicyanamido)­bis­(1,2,4-triazin-3-amine)­cobalt]; Şwitlicka-Olszewska *et al.*, 2016 ▸}.

## Synthesis and crystallization   

The title compound was prepared by adding a hot methano­lic solution (20 ml) of 3-amino-1,2,4-triazine (0.043g) (Aldrich) to a hot methano­lic solution (20 ml) of Nd(NO_3_)_3_·6H_2_O (0.219g) (Alfa Aesar). Di­chloro­methane (5 ml) was then added and the mixture refluxed for 7 h at 353 K. The resulting solution was then allowed to cool slowly to room temperature. After two weeks, brown-coloured crystals were obtained, m.p. = 378 K.

## Refinement details   

Crystal data, data collection and structure refinement details are summarized in Table 3 ▸. C-bound H atoms were placed geometrically and refined using the riding-model approximation: C—H = 0.93 Å with *U*_iso_(H) set to 1.2–1.5*U*_eq_(C). The water and N-bound H atoms were located in difference-Fourier maps and refined with *U*_iso_(H) = 1.2*U*_eq_(O) or 1.2*U*_eq_(N).

## Supplementary Material

Crystal structure: contains datablock(s) global, I. DOI: 10.1107/S2056989018011714/cq2026sup1.cif

Structure factors: contains datablock(s) I. DOI: 10.1107/S2056989018011714/cq2026Isup2.hkl

CCDC reference: 1583097

Additional supporting information:  crystallographic information; 3D view; checkCIF report

## Figures and Tables

**Table 1:** Selected bond lengths (Å)

Nd1—O1*A*	2.5876 (15)	Nd1—O4*B*	2.5698 (17)
Nd1—O1*W*	2.4826 (17)	Nd1—O4*W*	2.4540 (14)
Nd1—O2*A*	2.5480 (16)	Nd1—O5*B*	2.6402 (17)
Nd1—O2*W*	2.4603 (18)	Nd1—O7*C*	2.5428 (15)
Nd1—O3*W*	2.4790 (15)	Nd1—O8*C*	2.6161 (15)

**Table 2:** Hydrogen-bond geometry (Å, °)

*D*—H⋯*A*	*D*—H	H⋯*A*	*D*⋯*A*	*D*—H⋯*A*
O1*W*—H1*A*⋯N4*A* ^i^	0.79 (3)	2.09 (3)	2.876 (2)	179 (4)
O1*W*—H1*B*⋯N2*C* ^ii^	0.76 (3)	2.16 (3)	2.899 (3)	167 (3)
O2*W*—H2*A*⋯O6*B* ^iii^	0.67 (3)	2.14 (3)	2.791 (3)	168 (3)
O2*W*—H2*B*⋯N2*B* ^iv^	0.81 (3)	2.01 (3)	2.806 (3)	166 (3)
O3*W*—H3*A*⋯O7*C* ^v^	0.83 (3)	2.04 (3)	2.864 (2)	173 (3)
O3*W*—H3*B*⋯N4*B*	0.82 (3)	2.02 (3)	2.832 (3)	169 (3)
O4*W*—H4*A*⋯N4*C* ^vi^	0.82 (2)	2.05 (3)	2.871 (3)	172 (3)
O4*W*—H4*B*⋯N2*A*	0.84 (2)	2.00 (2)	2.829 (2)	170 (2)
N3*A*—H1*NA*⋯O2*A*	0.84 (2)	2.06 (2)	2.883 (2)	168 (2)
N3*C*—H2*NC*⋯N1*A* ^vii^	0.85 (2)	2.10 (2)	2.916 (3)	163 (2)
N3*A*—H2*NA*⋯N1*C* ^iii^	0.85 (2)	2.12 (2)	2.931 (3)	161 (2)
N3*C*—H1*NC*⋯O8*C* ^ii^	0.83 (2)	2.17 (3)	2.980 (3)	164 (3)
N3*B*—H1*NB*⋯O1*A*	0.83 (2)	2.17 (2)	2.992 (3)	171 (2)
N3*B*—H2*NB*⋯O9*C* ^vii^	0.84 (2)	2.46 (3)	3.046 (3)	128 (2)
C3*A*—H3*AA*⋯N1*B* ^viii^	0.93	2.60	3.245 (3)	127
C3*B*—H3*BA*⋯O6*B* ^ix^	0.93	2.58	3.475 (3)	161
C3*C*—H3*CA*⋯O4*B* ^vii^	0.93	2.54	3.328 (3)	142

Symmetry codes: (i) 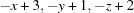; (ii) 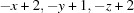; (iii) 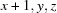; (iv) 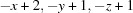; (v) 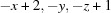; (vi) 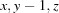; (vii) 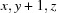; (viii) 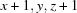; (ix) 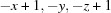.

**Table 3:** Experimental details

Crystal data	
Chemical formula	[Nd(NO_3_)_3_(H_2_O)_4_]·3C_3_H_4_N_4_
*M*_r_	690.64
Crystal system, space group	Triclinic, *P* 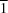
Temperature (K)	293
*a*, *b*, *c* (Å)	8.0279 (5), 10.8496 (5), 15.1239 (8)
α, β, γ (°)	102.228 (2), 96.148 (2), 102.764 (2)
*V* (Å^3^)	1239.11 (12)
*Z*	2
Radiation type	Mo *K*α
μ (mm^−1^)	2.18
Crystal size (mm)	0.35 × 0.30 × 0.30

Data collection	
Diffractometer	Bruker Kappa APEXII CCD
Absorption correction	Multi-scan (*SADABS*; Bruker, 2004 ▸)
*T*_min_, *T*_max_	0.517, 0.562
No. of measured, independent and observed [*I* > 2σ(*I*)] reflections	10023, 6016, 5620
*R*_int_	0.014
(sin θ/λ)_max_ (Å^−1^)	0.667

Refinement	
*R*[*F* ^2^ > 2σ(*F* ^2^)], *wR*(*F* ^2^), *S*	0.019, 0.047, 1.05
No. of reflections	6016
No. of parameters	399
No. of restraints	15
H-atom treatment	H atoms treated by a mixture of independent and constrained refinement
Δρ_max_, Δρ_min_ (e Å^−3^)	0.48, −0.41

Computer programs: *APEX2* (Bruker, 2004 ▸), *APEX2*, *SAINT* and *XPREP* (Bruker, 2004 ▸), *SIR92* (Altomare *et al.*, 1993 ▸), *SHELXL97* (Sheldrick, 2008 ▸) and *PLATON* (Spek, 2009 ▸).

## supplementary crystallographic information

### Crystal data

**Table:**

[Nd(NO_3_)_3_(H_2_O)_4_]·3C_3_H_4_N_4_	*Z* = 2
*M**_r_* = 690.64	*F*(000) = 686
Triclinic, *P*1	*D*_x_ = 1.851 Mg m^−^^3^
Hall symbol: -P 1	Mo *K*α radiation, λ = 0.71073 Å
*a* = 8.0279 (5) Å	Cell parameters from 7537 reflections
*b* = 10.8496 (5) Å	θ = 5.3–56.6°
*c* = 15.1239 (8) Å	µ = 2.18 mm^−^^1^
α = 102.228 (2)°	*T* = 293 K
β = 96.148 (2)°	Block, brown
γ = 102.764 (2)°	0.35 × 0.30 × 0.30 mm
*V* = 1239.11 (12) Å^3^	

### Data collection

**Table:**

Bruker Kappa APEXII CCD diffractometer	6016 independent reflections
Radiation source: fine-focus sealed tube	5620 reflections with *I* > 2σ(*I*)
Graphite monochromator	*R*_int_ = 0.014
Detector resolution: 18.4 pixels mm^-1^	θ_max_ = 28.3°, θ_min_ = 2.9°
ω and φ scan	*h* = −10→6
Absorption correction: multi-scan (SADABS; Bruker, 2004)	*k* = −14→14
*T*_min_ = 0.517, *T*_max_ = 0.562	*l* = −17→20
10023 measured reflections	

### Refinement

**Table:**

Refinement on *F*^2^	Primary atom site location: structure-invariant direct methods
Least-squares matrix: full	Secondary atom site location: difference Fourier map
*R*[*F*^2^ > 2σ(*F*^2^)] = 0.019	Hydrogen site location: inferred from neighbouring sites
*wR*(*F*^2^) = 0.047	H atoms treated by a mixture of independent and constrained refinement
*S* = 1.05	*w* = 1/[σ^2^(*F*_o_^2^) + (0.0271*P*)^2^ + 0.3994*P*] where *P* = (*F*_o_^2^ + 2*F*_c_^2^)/3
6016 reflections	(Δ/σ)_max_ = 0.001
399 parameters	Δρ_max_ = 0.48 e Å^−^^3^
15 restraints	Δρ_min_ = −0.41 e Å^−^^3^

### Special details

**Table:**

Geometry. Bond distances, angles etc. have been calculated using the rounded fractional coordinates. All su's are estimated from the variances of the (full) variance-covariance matrix. The cell esds are taken into account in the estimation of distances, angles and torsion angles
Refinement. Refinement on F^2^ for ALL reflections except those flagged by the user for potential systematic errors. Weighted R-factors wR and all goodnesses of fit S are based on F^2^, conventional R-factors R are based on F, with F set to zero for negative F^2^. The observed criterion of F^2^ > 2sigma(F^2^) is used only for calculating -R-factor-obs etc. and is not relevant to the choice of reflections for refinement. R-factors based on F^2^ are statistically about twice as large as those based on F, and R-factors based on ALL data will be even larger.

### Fractional atomic coordinates and isotropic or equivalent isotropic displacement parameters (Å^2^)

**Table:**

	*x*	*y*	*z*	*U*_iso_*/*U*_eq_	
Nd1	1.03611 (1)	0.21335 (1)	0.71016 (1)	0.0226 (1)	
O1A	1.0327 (2)	0.43232 (14)	0.66866 (10)	0.0403 (5)	
O1W	1.2955 (2)	0.21178 (17)	0.81482 (11)	0.0411 (5)	
O2A	1.1864 (3)	0.44707 (14)	0.79616 (10)	0.0488 (6)	
O2W	1.2906 (2)	0.26993 (18)	0.63563 (13)	0.0411 (6)	
O3A	1.1817 (2)	0.62147 (14)	0.74931 (13)	0.0521 (6)	
O3W	0.9189 (2)	0.18292 (17)	0.54640 (10)	0.0400 (5)	
O4B	0.7358 (2)	0.05414 (15)	0.68162 (12)	0.0433 (5)	
O4W	0.9528 (2)	0.25477 (15)	0.86308 (9)	0.0374 (5)	
O5B	0.7217 (2)	0.24915 (14)	0.68278 (11)	0.0396 (5)	
O6B	0.4922 (2)	0.09045 (19)	0.63361 (18)	0.0732 (8)	
O7C	1.0658 (2)	−0.00476 (14)	0.62008 (10)	0.0376 (5)	
O8C	1.0185 (2)	−0.00715 (14)	0.75724 (9)	0.0367 (5)	
O9C	1.0158 (4)	−0.18660 (17)	0.66249 (15)	0.0796 (8)	
N4	1.1347 (2)	0.50519 (15)	0.73869 (11)	0.0309 (5)	
N5	0.6465 (2)	0.13104 (17)	0.66539 (13)	0.0376 (6)	
N6	1.0336 (3)	−0.06974 (17)	0.68007 (12)	0.0377 (6)	
N1A	1.1227 (3)	0.36263 (18)	1.09013 (13)	0.0401 (6)	
N2A	1.1636 (2)	0.42269 (17)	1.02436 (11)	0.0368 (5)	
N3A	1.3275 (3)	0.5930 (2)	0.98173 (13)	0.0569 (7)	
N4A	1.3919 (2)	0.58214 (16)	1.13098 (11)	0.0333 (5)	
C1A	1.2941 (3)	0.53168 (18)	1.04680 (13)	0.0314 (6)	
C2A	1.2103 (3)	0.4099 (2)	1.17336 (15)	0.0403 (7)	
C3A	1.3477 (3)	0.5199 (2)	1.19349 (14)	0.0368 (6)	
N1B	0.4862 (3)	0.4080 (2)	0.36226 (14)	0.0455 (7)	
N2B	0.6180 (2)	0.49570 (18)	0.41878 (13)	0.0385 (6)	
N3B	0.8421 (3)	0.5452 (2)	0.53804 (15)	0.0445 (7)	
N4B	0.7041 (3)	0.32938 (17)	0.48005 (13)	0.0390 (6)	
C1B	0.7185 (3)	0.45541 (19)	0.47817 (14)	0.0308 (6)	
C2B	0.4607 (3)	0.2855 (3)	0.36603 (18)	0.0499 (8)	
C3B	0.5728 (4)	0.2461 (2)	0.42432 (18)	0.0490 (8)	
N1C	0.5817 (3)	0.82519 (19)	0.96809 (14)	0.0469 (7)	
N2C	0.7014 (3)	0.91061 (18)	1.03239 (13)	0.0418 (6)	
N3C	0.8979 (3)	1.1069 (2)	1.07868 (14)	0.0540 (7)	
N4C	0.7495 (3)	1.05383 (18)	0.93164 (12)	0.0409 (6)	
C1C	0.7815 (3)	1.02272 (19)	1.01297 (14)	0.0345 (6)	
C2C	0.5443 (4)	0.8506 (2)	0.88785 (17)	0.0522 (8)	
C3C	0.6296 (4)	0.9664 (3)	0.87013 (16)	0.0513 (8)	
H1A	1.381 (4)	0.268 (3)	0.8291 (18)	0.048 (8)*	
H1B	1.287 (4)	0.170 (3)	0.849 (2)	0.058 (9)*	
H2A	1.348 (3)	0.234 (3)	0.6331 (18)	0.034 (8)*	
H2B	1.327 (4)	0.331 (3)	0.614 (2)	0.069 (10)*	
H3A	0.924 (4)	0.126 (3)	0.5008 (19)	0.048 (7)*	
H3B	0.864 (4)	0.234 (3)	0.5329 (19)	0.058 (9)*	
H4A	0.903 (4)	0.199 (2)	0.8872 (19)	0.070 (10)*	
H4B	1.018 (3)	0.311 (2)	0.9070 (14)	0.045 (7)*	
H2AA	1.17990	0.36910	1.21960	0.0480*	
H3AA	1.40980	0.55000	1.25270	0.0440*	
H1NA	1.277 (3)	0.558 (2)	0.9278 (12)	0.050 (8)*	
H2NA	1.408 (3)	0.662 (2)	0.9910 (17)	0.054 (8)*	
H2BA	0.36580	0.22400	0.32900	0.0600*	
H3BA	0.55380	0.15820	0.42360	0.0590*	
H1NB	0.899 (3)	0.522 (2)	0.5781 (16)	0.056 (8)*	
H2NB	0.852 (4)	0.6243 (18)	0.5396 (19)	0.058 (8)*	
H3CA	0.60120	0.98250	0.81320	0.0620*	
H2CA	0.46000	0.79050	0.84290	0.0630*	
H2NC	0.944 (3)	1.1835 (18)	1.0753 (17)	0.051 (8)*	
H1NC	0.922 (4)	1.094 (3)	1.1302 (14)	0.057 (8)*	

### Atomic displacement parameters (Å^2^)

**Table:**

	*U*^11^	*U*^22^	*U*^33^	*U*^12^	*U*^13^	*U*^23^
Nd1	0.0279 (1)	0.0191 (1)	0.0187 (1)	0.0026 (1)	0.0010 (1)	0.0052 (1)
O1A	0.0466 (10)	0.0316 (7)	0.0363 (8)	0.0040 (7)	−0.0098 (7)	0.0088 (6)
O1W	0.0354 (9)	0.0432 (9)	0.0391 (9)	−0.0078 (7)	−0.0091 (7)	0.0239 (8)
O2A	0.0760 (13)	0.0296 (7)	0.0301 (8)	0.0018 (8)	−0.0119 (8)	0.0061 (6)
O2W	0.0373 (9)	0.0416 (9)	0.0584 (11)	0.0173 (8)	0.0195 (8)	0.0289 (8)
O3A	0.0621 (12)	0.0221 (7)	0.0650 (11)	0.0003 (7)	0.0025 (9)	0.0102 (7)
O3W	0.0592 (11)	0.0419 (9)	0.0220 (7)	0.0289 (8)	−0.0022 (7)	0.0018 (7)
O4B	0.0344 (9)	0.0343 (8)	0.0657 (11)	0.0100 (7)	0.0055 (7)	0.0214 (8)
O4W	0.0438 (9)	0.0375 (8)	0.0214 (7)	−0.0074 (7)	0.0051 (6)	0.0050 (6)
O5B	0.0430 (9)	0.0302 (7)	0.0447 (9)	0.0103 (6)	0.0091 (7)	0.0053 (7)
O6B	0.0275 (10)	0.0495 (11)	0.136 (2)	0.0093 (8)	−0.0057 (11)	0.0178 (12)
O7C	0.0561 (10)	0.0297 (7)	0.0311 (7)	0.0154 (7)	0.0109 (7)	0.0097 (6)
O8C	0.0503 (10)	0.0314 (7)	0.0274 (7)	0.0086 (7)	0.0002 (6)	0.0095 (6)
O9C	0.149 (2)	0.0259 (9)	0.0656 (13)	0.0272 (11)	0.0117 (14)	0.0119 (9)
N4	0.0360 (10)	0.0243 (8)	0.0300 (8)	0.0028 (7)	0.0066 (7)	0.0055 (7)
N5	0.0319 (10)	0.0344 (9)	0.0490 (11)	0.0099 (8)	0.0088 (8)	0.0127 (8)
N6	0.0503 (12)	0.0267 (8)	0.0343 (9)	0.0114 (8)	−0.0023 (8)	0.0064 (7)
N1A	0.0435 (11)	0.0342 (9)	0.0362 (10)	−0.0060 (8)	0.0033 (8)	0.0125 (8)
N2A	0.0416 (11)	0.0311 (9)	0.0280 (8)	−0.0076 (8)	−0.0007 (7)	0.0067 (7)
N3A	0.0721 (16)	0.0466 (12)	0.0277 (10)	−0.0302 (11)	−0.0059 (10)	0.0107 (9)
N4A	0.0353 (10)	0.0287 (8)	0.0273 (8)	−0.0020 (7)	−0.0029 (7)	0.0028 (7)
C1A	0.0346 (11)	0.0268 (9)	0.0258 (9)	−0.0017 (8)	0.0022 (8)	0.0026 (8)
C2A	0.0486 (14)	0.0388 (11)	0.0323 (11)	0.0026 (10)	0.0051 (9)	0.0158 (9)
C3A	0.0432 (13)	0.0350 (10)	0.0262 (10)	0.0039 (9)	−0.0023 (8)	0.0049 (8)
N1B	0.0407 (11)	0.0585 (12)	0.0442 (11)	0.0157 (9)	0.0051 (9)	0.0248 (10)
N2B	0.0398 (11)	0.0408 (10)	0.0434 (10)	0.0136 (8)	0.0100 (8)	0.0230 (9)
N3B	0.0446 (12)	0.0340 (10)	0.0529 (13)	0.0113 (9)	0.0010 (9)	0.0080 (9)
N4B	0.0484 (12)	0.0318 (9)	0.0400 (10)	0.0139 (8)	0.0025 (8)	0.0139 (8)
C1B	0.0332 (11)	0.0311 (9)	0.0337 (10)	0.0125 (8)	0.0118 (8)	0.0120 (8)
C2B	0.0440 (15)	0.0515 (14)	0.0479 (14)	0.0046 (11)	−0.0021 (11)	0.0112 (12)
C3B	0.0580 (16)	0.0323 (11)	0.0544 (15)	0.0064 (11)	0.0023 (12)	0.0139 (11)
N1C	0.0499 (13)	0.0376 (10)	0.0443 (11)	−0.0094 (9)	0.0066 (9)	0.0127 (9)
N2C	0.0441 (11)	0.0355 (9)	0.0418 (10)	−0.0064 (8)	0.0036 (8)	0.0193 (8)
N3C	0.0659 (15)	0.0432 (11)	0.0392 (11)	−0.0177 (10)	−0.0091 (10)	0.0223 (9)
N4C	0.0496 (12)	0.0372 (9)	0.0314 (9)	−0.0029 (8)	0.0046 (8)	0.0140 (8)
C1C	0.0371 (12)	0.0316 (10)	0.0329 (10)	−0.0003 (9)	0.0058 (8)	0.0133 (8)
C2C	0.0560 (16)	0.0444 (13)	0.0403 (13)	−0.0134 (12)	−0.0004 (11)	0.0076 (11)
C3C	0.0609 (17)	0.0527 (14)	0.0317 (11)	−0.0053 (12)	0.0009 (11)	0.0156 (11)

### Geometric parameters (Å, º)

**Table:**

Nd1—O1A	2.5876 (15)	N3A—C1A	1.318 (3)
Nd1—O1W	2.4826 (17)	N4A—C1A	1.356 (3)
Nd1—O2A	2.5480 (16)	N4A—C3A	1.309 (3)
Nd1—O2W	2.4603 (18)	C2A—C3A	1.388 (3)
Nd1—O3W	2.4790 (15)	N3A—H1NA	0.839 (18)
Nd1—O4B	2.5698 (17)	N3A—H2NA	0.85 (2)
Nd1—O4W	2.4540 (14)	N1B—C2B	1.314 (4)
Nd1—O5B	2.6402 (17)	N1B—N2B	1.331 (3)
Nd1—O7C	2.5428 (15)	C2A—H2AA	0.9300
Nd1—O8C	2.6161 (15)	N2B—C1B	1.348 (3)
O1A—N4	1.261 (2)	C3A—H3AA	0.9300
O2A—N4	1.263 (2)	N3B—C1B	1.327 (3)
O3A—N4	1.204 (2)	N4B—C1B	1.353 (3)
O4B—N5	1.258 (2)	N4B—C3B	1.305 (3)
O5B—N5	1.248 (2)	C2B—C3B	1.389 (4)
O6B—N5	1.225 (3)	N3B—H1NB	0.83 (2)
O7C—N6	1.276 (2)	N3B—H2NB	0.84 (2)
O8C—N6	1.253 (2)	N1C—C2C	1.319 (3)
O9C—N6	1.212 (3)	N1C—N2C	1.324 (3)
O1W—H1A	0.79 (3)	C2B—H2BA	0.9300
O1W—H1B	0.76 (3)	N2C—C1C	1.351 (3)
O2W—H2A	0.67 (3)	C3B—H3BA	0.9300
O2W—H2B	0.81 (3)	N3C—C1C	1.317 (3)
O3W—H3A	0.83 (3)	N4C—C1C	1.356 (3)
O3W—H3B	0.82 (3)	N4C—C3C	1.313 (3)
O4W—H4A	0.82 (2)	C2C—C3C	1.386 (4)
O4W—H4B	0.84 (2)	N3C—H2NC	0.85 (2)
N1A—N2A	1.331 (3)	N3C—H1NC	0.83 (2)
N1A—C2A	1.311 (3)	C2C—H2CA	0.9300
N2A—C1A	1.348 (3)	C3C—H3CA	0.9300
			
O1A—Nd1—O1W	116.22 (6)	H3A—O3W—H3B	112 (3)
O1A—Nd1—O2A	49.01 (5)	O1A—N4—O2A	115.11 (16)
O1A—Nd1—O2W	73.35 (6)	O2A—N4—O3A	122.41 (17)
O1A—Nd1—O3W	67.84 (5)	O1A—N4—O3A	122.47 (17)
O1A—Nd1—O4B	113.84 (5)	Nd1—O4W—H4B	120.9 (16)
O1A—Nd1—O4W	100.47 (5)	H4A—O4W—H4B	104 (2)
O1A—Nd1—O5B	67.01 (5)	Nd1—O4W—H4A	125.6 (19)
O1A—Nd1—O7C	133.51 (5)	O4B—N5—O5B	117.28 (17)
O1A—Nd1—O8C	175.74 (5)	O5B—N5—O6B	121.91 (19)
O1W—Nd1—O2A	71.32 (6)	O4B—N5—O6B	120.81 (19)
O1W—Nd1—O2W	72.07 (6)	O7C—N6—O9C	121.41 (19)
O1W—Nd1—O3W	142.96 (5)	O8C—N6—O9C	121.8 (2)
O1W—Nd1—O4B	126.63 (6)	O7C—N6—O8C	116.75 (17)
O1W—Nd1—O4W	75.68 (5)	N2A—N1A—C2A	119.5 (2)
O1W—Nd1—O5B	150.73 (5)	N1A—N2A—C1A	117.99 (17)
O1W—Nd1—O7C	85.26 (5)	C1A—N4A—C3A	115.25 (18)
O1W—Nd1—O8C	65.98 (5)	N2A—C1A—N3A	117.07 (19)
O2A—Nd1—O2W	75.74 (7)	N2A—C1A—N4A	124.71 (18)
O2A—Nd1—O3W	115.37 (5)	N3A—C1A—N4A	118.2 (2)
O2A—Nd1—O4B	138.98 (7)	N1A—C2A—C3A	121.0 (2)
O2A—Nd1—O4W	71.11 (5)	N4A—C3A—C2A	121.43 (19)
O2A—Nd1—O5B	96.55 (6)	C1A—N3A—H1NA	119.2 (15)
O2A—Nd1—O7C	147.45 (7)	C1A—N3A—H2NA	122.1 (17)
O2A—Nd1—O8C	131.19 (5)	H1NA—N3A—H2NA	118 (2)
O2W—Nd1—O3W	74.65 (6)	N2B—N1B—C2B	118.6 (2)
O2W—Nd1—O4B	141.46 (6)	N1A—C2A—H2AA	119.00
O2W—Nd1—O4W	139.48 (6)	C3A—C2A—H2AA	119.00
O2W—Nd1—O5B	132.07 (6)	N1B—N2B—C1B	118.67 (19)
O2W—Nd1—O7C	75.71 (6)	C2A—C3A—H3AA	119.00
O2W—Nd1—O8C	110.91 (6)	N4A—C3A—H3AA	119.00
O3W—Nd1—O4B	74.02 (6)	C1B—N4B—C3B	115.0 (2)
O3W—Nd1—O4W	141.32 (5)	N2B—C1B—N3B	117.8 (2)
O3W—Nd1—O5B	66.28 (5)	N2B—C1B—N4B	124.5 (2)
O3W—Nd1—O7C	71.06 (5)	N3B—C1B—N4B	117.6 (2)
O3W—Nd1—O8C	112.86 (5)	N1B—C2B—C3B	121.2 (2)
O4B—Nd1—O4W	78.33 (5)	N4B—C3B—C2B	121.7 (2)
O4B—Nd1—O5B	48.48 (5)	C1B—N3B—H1NB	118.5 (16)
O4B—Nd1—O7C	73.32 (5)	C1B—N3B—H2NB	120 (2)
O4B—Nd1—O8C	62.95 (5)	H1NB—N3B—H2NB	121 (3)
O4W—Nd1—O5B	75.18 (5)	N2C—N1C—C2C	120.0 (2)
O4W—Nd1—O7C	125.40 (5)	N1B—C2B—H2BA	119.00
O4W—Nd1—O8C	76.33 (5)	C3B—C2B—H2BA	119.00
O5B—Nd1—O7C	114.18 (5)	N1C—N2C—C1C	118.01 (19)
O5B—Nd1—O8C	109.20 (5)	C2B—C3B—H3BA	119.00
O7C—Nd1—O8C	49.31 (5)	N4B—C3B—H3BA	119.00
Nd1—O1A—N4	96.98 (11)	C1C—N4C—C3C	115.1 (2)
Nd1—O2A—N4	98.85 (12)	N2C—C1C—N3C	116.8 (2)
Nd1—O4B—N5	98.07 (12)	N2C—C1C—N4C	124.7 (2)
Nd1—O5B—N5	94.94 (11)	N3C—C1C—N4C	118.6 (2)
Nd1—O7C—N6	98.21 (12)	N1C—C2C—C3C	120.5 (2)
Nd1—O8C—N6	95.31 (11)	N4C—C3C—C2C	121.7 (2)
Nd1—O1W—H1B	120 (2)	C1C—N3C—H2NC	123.1 (17)
H1A—O1W—H1B	111 (3)	C1C—N3C—H1NC	123 (2)
Nd1—O1W—H1A	125 (2)	H2NC—N3C—H1NC	113 (3)
H2A—O2W—H2B	107 (3)	N1C—C2C—H2CA	120.00
Nd1—O2W—H2A	121 (3)	C3C—C2C—H2CA	120.00
Nd1—O2W—H2B	132 (2)	N4C—C3C—H3CA	119.00
Nd1—O3W—H3B	118 (2)	C2C—C3C—H3CA	119.00
Nd1—O3W—H3A	130 (2)		
			
O1W—Nd1—O1A—N4	−24.39 (13)	O1W—Nd1—O8C—N6	−109.08 (14)
O2A—Nd1—O1A—N4	1.33 (11)	O2A—Nd1—O8C—N6	−140.23 (14)
O2W—Nd1—O1A—N4	−84.04 (12)	O2W—Nd1—O8C—N6	−51.15 (15)
O3W—Nd1—O1A—N4	−163.87 (13)	O3W—Nd1—O8C—N6	30.42 (15)
O4B—Nd1—O1A—N4	136.46 (11)	O4B—Nd1—O8C—N6	87.01 (14)
O4W—Nd1—O1A—N4	54.70 (12)	O4W—Nd1—O8C—N6	170.72 (14)
O5B—Nd1—O1A—N4	123.53 (12)	O5B—Nd1—O8C—N6	102.04 (14)
O7C—Nd1—O1A—N4	−134.30 (11)	O7C—Nd1—O8C—N6	−3.78 (13)
O1A—Nd1—O2A—N4	−1.33 (11)	Nd1—O1A—N4—O2A	−2.24 (19)
O1W—Nd1—O2A—N4	154.40 (15)	Nd1—O1A—N4—O3A	176.58 (16)
O2W—Nd1—O2A—N4	78.84 (14)	Nd1—O2A—N4—O1A	2.28 (19)
O3W—Nd1—O2A—N4	13.85 (16)	Nd1—O2A—N4—O3A	−176.53 (16)
O4B—Nd1—O2A—N4	−80.79 (15)	Nd1—O4B—N5—O5B	−11.4 (2)
O4W—Nd1—O2A—N4	−124.82 (15)	Nd1—O4B—N5—O6B	168.5 (2)
O5B—Nd1—O2A—N4	−52.97 (14)	Nd1—O5B—N5—O4B	10.98 (19)
O7C—Nd1—O2A—N4	108.17 (15)	Nd1—O5B—N5—O6B	−168.9 (2)
O8C—Nd1—O2A—N4	−175.68 (11)	Nd1—O7C—N6—O8C	−6.6 (2)
O1A—Nd1—O4B—N5	−9.63 (14)	Nd1—O7C—N6—O9C	172.2 (3)
O1W—Nd1—O4B—N5	148.87 (12)	Nd1—O8C—N6—O7C	6.4 (2)
O2A—Nd1—O4B—N5	44.60 (16)	Nd1—O8C—N6—O9C	−172.4 (3)
O2W—Nd1—O4B—N5	−102.61 (14)	C2A—N1A—N2A—C1A	−0.6 (3)
O3W—Nd1—O4B—N5	−65.88 (13)	N2A—N1A—C2A—C3A	−1.5 (4)
O4W—Nd1—O4B—N5	86.78 (13)	N1A—N2A—C1A—N3A	−177.0 (2)
O5B—Nd1—O4B—N5	6.34 (11)	N1A—N2A—C1A—N4A	2.8 (3)
O7C—Nd1—O4B—N5	−140.42 (13)	C3A—N4A—C1A—N2A	−2.5 (3)
O8C—Nd1—O4B—N5	167.26 (14)	C3A—N4A—C1A—N3A	177.3 (2)
O1A—Nd1—O5B—N5	157.78 (13)	C1A—N4A—C3A—C2A	0.2 (3)
O1W—Nd1—O5B—N5	−99.23 (15)	N1A—C2A—C3A—N4A	1.8 (4)
O2A—Nd1—O5B—N5	−162.21 (12)	C2B—N1B—N2B—C1B	−0.7 (3)
O2W—Nd1—O5B—N5	121.11 (12)	N2B—N1B—C2B—C3B	−3.1 (4)
O3W—Nd1—O5B—N5	82.92 (12)	N1B—N2B—C1B—N3B	−175.6 (2)
O4B—Nd1—O5B—N5	−6.36 (11)	N1B—N2B—C1B—N4B	5.5 (3)
O4W—Nd1—O5B—N5	−93.77 (12)	C3B—N4B—C1B—N2B	−5.9 (4)
O7C—Nd1—O5B—N5	28.78 (13)	C3B—N4B—C1B—N3B	175.3 (2)
O8C—Nd1—O5B—N5	−24.32 (13)	C1B—N4B—C3B—C2B	1.8 (4)
O1A—Nd1—O7C—N6	−171.95 (13)	N1B—C2B—C3B—N4B	2.6 (4)
O1W—Nd1—O7C—N6	65.87 (14)	C2C—N1C—N2C—C1C	0.2 (4)
O2A—Nd1—O7C—N6	109.22 (15)	N2C—N1C—C2C—C3C	0.0 (4)
O2W—Nd1—O7C—N6	138.56 (14)	N1C—N2C—C1C—N3C	178.9 (2)
O3W—Nd1—O7C—N6	−143.06 (15)	N1C—N2C—C1C—N4C	−0.8 (4)
O4B—Nd1—O7C—N6	−64.65 (14)	C3C—N4C—C1C—N2C	0.9 (4)
O4W—Nd1—O7C—N6	−2.82 (16)	C3C—N4C—C1C—N3C	−178.7 (3)
O5B—Nd1—O7C—N6	−91.39 (14)	C1C—N4C—C3C—C2C	−0.6 (4)
O8C—Nd1—O7C—N6	3.74 (13)	N1C—C2C—C3C—N4C	0.2 (5)

### Hydrogen-bond geometry (Å, º)

**Table:**

*D*—H···*A*	*D*—H	H···*A*	*D*···*A*	*D*—H···*A*
O1*W*—H1*A*···N4*A*^i^	0.79 (3)	2.09 (3)	2.876 (2)	179 (4)
O1*W*—H1*B*···N2*C*^ii^	0.76 (3)	2.16 (3)	2.899 (3)	167 (3)
O2*W*—H2*A*···O6*B*^iii^	0.67 (3)	2.14 (3)	2.791 (3)	168 (3)
O2*W*—H2*B*···N2*B*^iv^	0.81 (3)	2.01 (3)	2.806 (3)	166 (3)
O3*W*—H3*A*···O7*C*^v^	0.83 (3)	2.04 (3)	2.864 (2)	173 (3)
O3*W*—H3*B*···N4*B*	0.82 (3)	2.02 (3)	2.832 (3)	169 (3)
O4*W*—H4*A*···N4*C*^vi^	0.82 (2)	2.05 (3)	2.871 (3)	172 (3)
O4*W*—H4*B*···N2*A*	0.84 (2)	2.00 (2)	2.829 (2)	170 (2)
N3*A*—H1*NA*···O2*A*	0.84 (2)	2.06 (2)	2.883 (2)	168 (2)
N3*C*—H2*NC*···N1*A*^vii^	0.85 (2)	2.10 (2)	2.916 (3)	163 (2)
N3*A*—H2*NA*···N1*C*^iii^	0.85 (2)	2.12 (2)	2.931 (3)	161 (2)
N3*C*—H1*NC*···O8*C*^ii^	0.83 (2)	2.17 (3)	2.980 (3)	164 (3)
N3*B*—H1*NB*···O1*A*	0.83 (2)	2.17 (2)	2.992 (3)	171 (2)
N3*B*—H2*NB*···O9*C*^vii^	0.84 (2)	2.46 (3)	3.046 (3)	128 (2)
C3*A*—H3*AA*···N1*B*^viii^	0.93	2.60	3.245 (3)	127
C3*B*—H3*BA*···O6*B*^ix^	0.93	2.58	3.475 (3)	161
C3*C*—H3*CA*···O4*B*^vii^	0.93	2.54	3.328 (3)	142

Symmetry codes: (i) −*x*+3, −*y*+1, −*z*+2; (ii) −*x*+2, −*y*+1, −*z*+2; (iii) *x*+1, *y*, *z*; (iv) −*x*+2, −*y*+1, −*z*+1; (v) −*x*+2, −*y*, −*z*+1; (vi) *x*, *y*−1, *z*; (vii) *x*, *y*+1, *z*; (viii) *x*+1, *y*, *z*+1; (ix) −*x*+1, −*y*, −*z*+1.
